# Can Medical Students Learn and Perform POCUS in the Pediatric Emergency Department? Implementation of a Short Curriculum

**DOI:** 10.24908/pocus.v7i1.15625

**Published:** 2022-04-21

**Authors:** Michael C Cooper, Jodi Jones, Mandy Pascual, Steven Field, Juan M Rendon, Christine Kulstad, Bryant Dixon, Kristie Pham Tu, Aman Narayan, Hunter Pyle, Khiem Hoang, Anthony Han, Dalbir Bahga, Aman Pandey, Lynn Roppolo

**Affiliations:** 1 Department of Pediatrics, Division of Emergency Medicine, University of Texas Southwestern USA; 2 Department of Emergency Medicine, University of Texas Southwestern USA; 3 Medical School, University of Texas Southwestern USA

**Keywords:** POCUS, clinical ultrasound, medical student

## Abstract

**Purpose: **To determine medical student ability to accurately obtain and interpret POCUS exams of varying difficulty in the pediatric population after a short didactic and hands-on POCUS course. **Methods: **Five medical students were trained in four POCUS applications (bladder volume, long bone for fracture, limited cardiac for left ventricular function, & inferior vena cava collapsibility) and enrolled pediatric ED patients. Ultrasound-fellowship-trained emergency medicine physicians reviewed each scan for image quality and interpretation accuracy using the American College of Emergency Physicians’ quality assessment scale. We report acceptable scan frequency and medical student vs. Ultrasound-fellowship-trained emergency medicine physician interpretation agreement with 95% confidence intervals (CI). **Results: **Ultrasound-fellowship-trained emergency medicine physicians graded 51/53 bladder volume scans as acceptable (96.2%; 95% CI 87.3-99.0%) and agreed with 50/53 bladder volume calculations (94.3%; 95% CI 88.1-100%). Ultrasound-fellowship-trained emergency medicine physicians graded 35/37 long bone scans as acceptable (94.6%; 95% CI 82.3-98.5%) and agreed with 32/37 medical student long bone scan interpretations (86.5%; 95% CI 72.0-94.1%). Ultrasound-fellowship-trained emergency medicine physicians graded 116/120 cardiac scans as acceptable (96.7%; 95% CI 91.7-98.7%) and agreed with 111/120 medical student left ventricular function interpretations (92.5%; 95% CI 86.4-96.0%). Ultrasound-fellowship-trained emergency medicine physicians graded 99/117 inferior vena cava scans as acceptable (84.6%; 95% CI 77.0-90.0%) and agreed with 101/117 medical student interpretations of inferior vena cava collapsibility (86.3%; 95% CI 78.9-91.4%). **Conclusions: **Medical students demonstrated satisfactory ability within a short period of time in a range of POCUS scans on pediatric patients after a novel curriculum. This supports the incorporation of a formal POCUS education into medical school curricula and suggests that novice POCUS learners can attain a measure of competency in multiple applications after a short training course.

## Introduction

Point-of-care ultrasound (POCUS) is used by emergency physicians to make rapid critical diagnoses in the emergency department (ED) 1. POCUS is now being incorporated into medical student patient assessment curricula 2, 3. Several studies have demonstrated the feasibility of medical students using POCUS and have included teaching multiple POCUS applications to medical students simultaneously 2, 4, 5. However, there are few studies demonstrating medical students’ ability to accurately perform POCUS on pediatric patients and these studies have typically included one POCUS application taught at a time 6, 7, 8, 9. 

POCUS is particularly well suited to aid the evaluation of pediatric patients 10. When it comes to POCUS, children are not little adults: their smaller body size, higher ratio of cartilage to bone and decreased fat to lean body mass are suitable to ultrasound. However, their fear of clinical interactions and lack of cooperation can make physical and POCUS exams more difficult, and it is often beneficial to utilize distraction technique to facilitate the exam 11. It has been demonstrated that POCUS training has a positive effect on medical student anatomic and physiologic knowledge, clinical decision making, and development of clinical skills 12, 13, 14. It has also been suggested that early medical student ultrasound training may prevent future diagnostic mistakes by maximizing their ability to obtain accurate ultrasound images 15. However, it has not been shown that medical students could achieve the level of competency needed to obtain and accurately interpret quality POCUS images in pediatric patients across a spectrum of POCUS exams.

Emergency medicine (EM) and pediatric EM ultrasound curricula generally teach a wide variety of POCUS scans, ranging from easy-to-master scans, such as bladder volume assessment, to moderately difficult scans, such as for long bone fractures, to more difficult scans, such as limited echocardiography and vascular scans. 

Bladder volume measured with the use of POCUS is more reliable than automated bladder scans 16. The two main benefits of teaching bladder volume scans to medical students include teaching an easy to learn scan, paving the way for more advanced applications, and teaching a useful clinical adjunct that can help decide the timing of bladder catheterization in young pediatric patients.

POCUS has been successfully used by emergency physicians to diagnose pediatric extremity fractures. A study by Barata^17^ demonstrated a high sensitivity (95.3%) and specificity (85.5%) for identifying suspected pediatric long bone fractures with ultrasound performed by physicians trained via a brief didactic session and video review of normal and fractured long-bones. This was similar to results from a study by Poonai^18^ showing a sensitivity of 94.7% and specificity of 93.5% in ultrasound performed by physicians with at least 2 years POCUS experience who also reviewed a video and performed 25 practice scans prior to the study. The minimal training and satisfactory results in both novice and experienced POCUS users suggest that it is an application that could be included in medical student POCUS curricula. 

Vomiting, diarrhea, and volume depletion are common presentations of pediatric ED patients. POCUS assessment of inferior vena cava (IVC) diameter and respiratory variation and left ventricular (LV) function have been used to determine responsiveness in clinically hypovolemic patients and in the assessment of unexplained hypotension or shock 19, 20, 21. The benefits of teaching IVC and cardiac POCUS to medical students include earlier detection of patients with clinically relevant volume depletion and shock. 

The purpose of this study was to demonstrate the ability of medical students with limited training in POCUS to accurately obtain and interpret ultrasound exams across a spectrum of exam difficulty in pediatric patients. For this study, we chose to examine medical students’ ability to perform POCUS examinations for bladder volume assessment, long bone fracture identification, LV function assessment, and IVC collapsibility in pediatric patients. 

## Methods

### Study Approval

This study was approved by the institutional review boards of the University of Texas Southwestern and Children’s Medical Center of Dallas. All subjects underwent informed consent in either English or Spanish. 

### Medical Student Recruitment and Training

Five medical students with no prior ultrasound experience were recruited from the medical student emergency medicine interest group and underwent informed consent to participate. Training was scheduled over the two weeks following the completion of their first year of medical school and included lectures in ultrasound physics, bladder assessment, long bone fracture assessment, limited echocardiography, and IVC assessment given by ultrasound-fellowship-trained emergency physicians (USEMP). The medical students also observed two 4-hour quality assurance (QA) sessions that were part of the EM resident ultrasound rotation which included image review of these ultrasound techniques and related topics. Medical students also received training in obtaining informed consent and hands on practice with volunteers on all US techniques in this study. The investigators also created a simulated bone model as all volunteers for the bone ultrasound did not have fractures. The students were required to complete one Standardized Direct Observational Tool (SDOT) of patient enrollment and study technique for each study application on a patient or medical student volunteer at the end of the training period with a faculty investigator. Any students with unsatisfactory completion of one or more steps completed additional SDOTs until all steps were completed satisfactorily.

### Patient Inclusion and Exclusion Criteria

Inclusion criteria for bladder ultrasound exam subjects included all patients under age 18 years requiring urethral catheterization with an English or Spanish speaking parent or guardian present. Exclusion criteria included patients unable to tolerate the POCUS exam and patients not consenting to participate.

Inclusion criteria for long bone ultrasound exams subjects included all patients under age 18 years presenting with traumatic extremity pain with an English or Spanish speaking parent or guardian. Exclusion criteria included patients unable to tolerate the POCUS exam, patients with concern for open fracture, patients with radiology department imaging already performed, and patients not consenting to participate.

Inclusion criteria for cardiac & IVC exams subjects were clinically stable ED patients under 14 years old with clinical euvolemia as determined by the treating physician (ED attending or pediatric EM fellow) with an English or Spanish speaking parent or guardian present. Exclusion included: 1. recent vomiting, diarrhea, or decreased oral intake, 2. clinical impression of volume depletion or shock, 3. Inability to lay supine for the study procedure, 4. patient history of congenital heart disease, renal disease, liver cirrhosis, heart transplant, liver transplant, Marfan syndrome, or complex care (e.g. tube feedings or parental nutrition), 5. patients not consenting to participate.

### Patient enrollment

Medical students staffed an urban tertiary pediatric ED 24 hours a day for 5 weeks in 12 hour shifts from late June - early August 2019. The medical student searched the ED track board for patients meeting inclusion criteria. The ED attending or fellow approved patients for study enrollment. The patient’s parent/guardian underwent informed consent via a standard script. Spanish speaking parents/guardians were consented in person with the hospital-based interpreter when available or via telephone by an on-call Spanish speaking study staff member. Patients over age 10 years gave assent to participate. 

### Ultrasound examination procedures

Patient demographics including age, sex, and ethnicity, as well as the medical student performing the scan were recorded. All scans were completed using a Mindray TE 7 ultrasound with a 2.9-10.5 mHz phased array transducer (younger patients undergoing IVC/cardiac/bladder scans) or 2-4 mHz phased array transducer (typically for older patients undergoing IVC/cardiac/bladder scans) or 2-8 mHz linear array transducer (long bone scans) as deemed appropriate by the medical student performing the scan. All relevant still images and clips were reviewed by ultrasound trained faculty.

Bladder volume study: Patients were placed supine. Still images of the bladder were obtained in longitudinal and transverse views pre- and post-catheterization. Anterior-posterior, transverse and head-to-toe measurements were obtained, and bladder volume was calculated by the ultrasound machine. 

Fracture study: Patients were placed in a position of optimal comfort. The ultrasound transducer was placed over the point of maximal tenderness (determined by patient indication in verbal patients and by treating physician exam in nonverbal patients) with enough ultrasound gel to limit contact of the transducer with the skin. Eight second video clips were obtained in longitudinal and transverse views in the area of interest. Location of pain, medical student interpretation of fracture presence or absence, and presence or absence of fracture on radiology department imaging, were recorded. 

Cardiac/IVC scans: Patients were placed supine. The medical student obtained 8 second ultrasound clips of the longitudinal IVC and parasternal long and apical 4 chamber cardiac views. medical student qualitative assessment of LV function (hyperdynamic, normal function or hypodynamic [mild, moderate or severe]) and IVC collapsibility (greater than or less than 50%) were recorded. 

### Review of ultrasound scans

Recorded information was entered into a REDCap database by three study team EM residents. Each clip and still image was securely downloaded by the Principal Investigator and given directly to the USEMP responsible for reviewing the images. Each scan was reviewed by one of four USEMPs for image quality and accuracy of medical student interpretation. Each scan was reviewed by a second USEMP if the first disagreed with the medical student interpretation. Quality was graded based on the American College of Emergency Physician’s (ACEP) emergency ultrasound standard reporting guidelines’ 5-point quality assurance (QA) scale with a score of 3 or above meeting minimum criteria for diagnosis. The scale is as follows:

1: No recognizable structure, no objective data can be gathered 

2: Minimally recognizable structures but insufficient for diagnosis 

3: Minimal criteria met for diagnosis, recognizable structures but with some technical or other flaws 

4: Minimal criteria met for diagnosis, all structures imaged well and diagnosis easily supported 

5: Minimal criteria met for diagnosis, all structures imaged with excellent image quality and diagnosis completely supported

### Data Analysis

All data from the REDCap database were transferred to Microsoft Excel 365. We report the frequency of acceptable scans obtained by the medical student as determined by the USEMP and the agreement between the medical student interpretation and the USEMP interpretation, both with 95% confidence intervals (CI).

## Results

Each medical student completed an average of 25.8 practice scans during the training period (Table 1). 

**Table 1:** Practice Scans Completed by Medical Students.

**Ultrasound exam**	**Average number of practice** **scans per medical student (Range)**
**Bladder**	5.2 (4-7)
**Bone**	4.4 (3-6)
**IVC**	9.4 (8-11)
**Cardiac**	6.8 (5-8)

During the study period, 323 scans on 210 ED patients age 1 week to 17 years (Table 2) were completed by the medical students. Each medical student performed between 22 to 143 scans. 

**Table 2:** Patient Demographics.

	**Bladder**	**Fracture**	**IVC/CV**
**Number of Patients**	53	37	120
**Age Range (mean years)**	4d – 15 yrs (1.37 yrs)	1.5 yrs – 17 yrs (8.5 yrs)	1 wk – 15 yrs (6.6 yrs)
**Male, # (%)**	22 (40%)	20 (51%)	64 (53.3%)
**Race, # (%)** · **African American** · **Caucasian** · **More than one** · **Unknown/not reported**	- 17 (31%) 26 (47%) 2 (4%) 10 (18%)	- 8 (20.5%) 25 (64.1%) 0 6 (15.4%)	- 36 (30%) 71 (59.1%) 1 (0.8%) 12 (10%)
**Hispanic, # (%)**	26 (47%)	21 (53.8%)	54 (45%)
**BMI range, (median)**	N/A	N/A	10.9-33 (17.7)
**Location of extremity pain, # (%)** **Upper arm** **Forearm** **Thigh** **Lower leg**	N/A	- - 5 (13.5 %) 11 (29.7 %) 8 (21.6 %) 13 (35.1 %)	N/A

### Bladder Volume

Fifty-three ED patients age 1 week to 15 years had bladder scans completed during the study. Two additional patients were approached and consented for enrollment but did not complete the study and were excluded from analysis. Each medical student performed an average of 10.6 bladder scans (range 3-17). The USEMP graded 51/53 scans as QA 3 or above (96.2%; 95% CI 87.3-99.0%). One scan of poor quality was graded as QA 2 by the USEMP, consistent with the student’s interpretation and the second USEMP. The second scan of poor quality was graded QA 1 by the USEMP, consistent with the student’s interpretation and the second USEMP. Bladder volumes were measured in 50/53 subjects and the USEMP agreed with all of the measurements submitted, yielding an accuracy of 94.3% based on bladder volume scans attempted. Of the 3 studies without volume measurements, the USEMP stated that 2 of the studies had inadequate images to calculate bladder volumes and that 1 of the studies appeared to have adequate images to calculate bladder volume. The medical students’ mean self-reported image quality was 3.25, while the faculty perception of students’ image quality was 4.10 (p<.001; Table 3).

**Table 3:** Percentage of acceptable scans and accuracy of interpretation of POCUS scans completed by Medical Students.

**Scan**	**QA ≥ 3 / Total (%)**	**Accurate medical student interpretation per USEMP (%)**
Bladder	51/53 (96.2%)	50/53 (94.3%)
Long Bone	35/37 (94.6%)	32/37 (86.5%)
Cardiac	116/120 (96.7%)	111/120 (92.5%)
IVC	99/117 (84.6%)	101/117 (86.3%)

Bladder catheterization volumes were available for 49 of the 53 patients undergoing bladder volume assessment. Four patients did not undergo in and out (I&O) catheterization. Bladder volume adequate for urinalysis and urine culture was defined as greater than or equal to 2.5 mL 22, 23. Of the 49 patients, 47 had bladder volumes that measured > 2.5 mL, and 42 of those patients had > 2.5 mL collected by I&O catheterization. Two patients had bladder volumes measured as less than 2.5 mL. One of those patients had a measured volume of 1.43 mL with 0.2 mL collected, the other had a measured volume of less than 1 mL with 5 mL collected. The POCUS bladder volume measurement of > 2.5 mL predicting adequate volume for I&O cath yielded a sensitivity of 97.7%, similar to prior studies in bladder volume measurement 22, 23, though the specificity was much lower at 16.7%. The measured bladder volumes in the I/O caths yielding inadequate urine volume were 5.82 mL, 7.4 mL, 10.34 mL, 10.54 mL, and 18.92 mL. The overall accuracy compared to I&O cath volume was 87.8%.

### Long Bone Scans for Presence/absence of Fracture

During the study, 37 ED patients aged 18 months to 17 years had long bone scans for the presence/absence of fracture (fracture scans) completed. An additional 2 patients were enrolled but did not complete the study and were excluded from analysis. Each medical student performed an average of 7.4 long bone scans (range 4-14). Medical students completed 5 humerus scans, 11 radius/ulna scans, 8 femur scans, and 13 tibia/fibula scans. USEMP graded 35/37 long bone scans as able to be interpreted with image quality QA 3 or above (94.6%; 95% CI 82.3-98.5%). USEMP agreed with 32/37 medical student interpretations (presence or absence of fracture) of long bone fracture scans (86.5%; 95% CI 72.0-94.1%). Of the 5 scans in which the USEMP did not agree with the medical student interpretation, both USEMP stated that 2 of the study images were of too poor quality to support an interpretation. On the three other scans in disagreement, the USMEP both stated that fractures were present on ultrasound when the medical student stated no fracture was present (Table 2).

Radiograph results were available for 35 of 37 patients who completed the fracture US scans. Medical students identified 12 of 16 fractures detected by radiograph and stated no fracture was present on 17 of 19 negative radiographs. This yielded a sensitivity of 75.0% (95% CI 47.6-92.7%), specificity of 89.5% (95% CI 66.9-98.7%), positive predictive value of 85.7% (95% CI 61.1-95.8%), and negative predictive value of 82.9% (95% CI 64.2-91.0%) when compared to radiographs, with an overall accuracy of 82.9% (95% CI 66.4-93.4%; Table 3).

### Cardiac 2 view: parasternal long axis and apical 4 chamber

Cardiac scans were completed in 120 ED patients age 1 week to 13 years. An additional 15 patients were enrolled but did not complete the study and were excluded from analysis. Each medical student performed an average of 24 cardiac scans (range 8-57). USEMP graded 116/120 cardiac scans 3 or above (96.7%; 95% CI 91.7-98.7%). USEMP agreed with medical student interpretation of LV function on 111/120 scans (92.5%; 95% CI 86.4-96.0%). In 3 of the 9 scans in which the USEMP did not agree with the medical student interpretation, the medical student stated there was moderately diminished LV function (1 scan) or mildly diminished function (2 scans) and both USEMP stated the LV function was normal. Of the other 6 scans in which there was disagreement, the medical student stated there was normal LV function whereas both faculty stated the images were of too poor quality to support an interpretation (Table 3). As there were no scans with diminished function by USEMP interpretation, sensitivity and positive predictive value for LV failure cannot be calculated. However, the medical students’ specificity was 97.3% and negative predictive value was 94.9%, with an accuracy of 92.5%.

### IVC

There were 117 ED patients, aged 1 week to 13 years who had IVC scans completed during the study. An additional 18 patients were enrolled but did not complete the study and were therefore excluded from analysis. Each medical student performed an average of 23.4 IVC scans (range 5-56). USEMP graded 99/117 IVC scans as a 3 or above (84.6%; 95% CI 77.0-90.0%). The USEMP agreed with 101/117 medical student interpretations of IVC collapsibility of greater or less than 50% (86.3%; 95% CI 78.9-91.4%; Table 3).

Of the 99 scans of sufficient quality for interpretation, the medical student and USEMP agreed on > 50% collapse in 16 subjects and agreed on < 50% collapse in 81 subjects. There were 2 patients that the medical student interpreted as > 50% collapse where the USEMP disagreed. There were no disagreements where the medical student stated < 50% collapse. This yielded a sensitivity of 100%, specificity of 88%, positive predictive value of 88.9%, and negative predictive value of 100%, with an accuracy of 98%. 

## Discussion

Medical schools are increasingly integrating ultrasound into their curriculum, which has been encouraged by the American Academy of Emergency Medicine 2, 24, 25, 26, 27, 28, 29. Teaching POCUS along with fundamental clinical examination skills may improve both skill sets 28. Multiple studies have shown that combined didactic and hands-on learning improves learning outcomes, and that medical students with limited training can identify pathologies and possibly affect patient outcomes 30, 31, 32. Udrea, et al., demonstrated that POCUS performed by second year medical students in an adult ED demonstrated a 94.7% agreement between treating physicians and the medical student performed studies and led to a newly discovered diagnosis in 12.4% of scans, changed the initial management plan in 17.3% of scans, reduced time to disposition 33.5% of the time, and led to an avoidance of additional imaging studies 53% of the time 33. However, despite the expanding use of POCUS and its incorporation into medical school education, there are few studies examining the diagnostic accuracy of medical student-performed POCUS 33, 34, 35.

In this study, we studied the proficiency achieved by medical students through a short ultrasound course covering clinical ultrasound scans of varying difficulty (similar to our approach in teaching EM residents during their clinical ultrasound rotation), comparing the diagnostic accuracy of the medical students’ interpretations with the USEMP’s QA review of the scan. Current expert guidelines for credentialing pediatric EM faculty in clinical ultrasound recommend a minimum of 5 bladder volume scans, 10 IVC scans, 25 musculoskeletal scans and 25 cardiac scans as part of a competency assessment 36, with an increased number of required scans reflecting a greater difficulty to achieve competency. 

Medical students demonstrated adequate competency in ultrasound acquisition with regard to the organ of interest with a QA grade 3 or better in 96.2% of bladder scans, 94.6% of long bone scans, 96.7% of cardiac scans, and 84.6% of IVC scans. Medical students also displayed a satisfactory ability to arrive at an accurate diagnosis compared to the gold standard USEMP interpretation of 94.3% for bladder scans (87.8% vs I&O cath volume), 86.5% for long bone scans (82.9% vs radiograph), 92.5% for cardiac scans, and 86.3% for attempted IVC scans (97.9% of interpretable IVC scans). The most likely reason for a USEMP to disagree with an medical student interpretation was an interpretation submitted for a scan of QA 1 or 2. We report the results in this format to give a more realistic view that scans should be of sufficient quality in order to make a diagnosis. The degree of accuracy is similar to a study by Andersen et al 35, who examined medical students’ use of pocket-sized POCUS devices on hospitalized adult patients after a POCUS curriculum in identification of LV function, pericardial effusion (on an apical 4-chamber view only), pleural effusion, lung comets, hydronephrosis, bladder distention, gallstones, abdominal free fluid, cholecystitis, and aorta and IVC diameters. Twenty-five medical students self-selected 1151 scans which were reviewed by unblinded radiologists or cardiologists. Given the self-selection bias, the scans were determined to have an acceptable organ presentation (similar to our QA > 3) in 73.8% of cardiovascular scans and 88.4% of radiologic scans, with a diagnostic accuracy of 93.5% of acceptable cardiac scans and 93.2% of acceptable radiologic scans. In our study, the overall diagnostic accuracy was similar despite the inclusion of nondiagnostic QA 1 or 2 scans. 

The medical students’ ability to rule-in or rule-out long bone fractures had a lower sensitivity of 75% but a specificity of 89.5%, PPV of 85.7% and NPV of 82.9%. A prior meta-analysis of long-bone fracture diagnosis by POCUS showed a pooled sensitivity of 64.7-100% and specificity of 79.2-100% placing the medical student performance in the current investigation well within the previously documented ranges of long bone fracture diagnosis with POCUS 37. Additional possibilities explaining the lower sensitivity of medical students to detect lone bone fractures may be that this was the least practiced scan during the practice scanning portion of the educational course, with medical students practicing on average 4.4 long bone scans vs 5.2 bladder scans, 6.8 LV cardiac scans, and 9.4 IVC scans. Additionally, lone bone scan practice was done on healthy volunteers, and the practice scans were all normal, at least suggesting that seeing pathology during training may be beneficial to the development of POCUS skills. 

It has been previously demonstrated that incorporation POCUS education into existing medical student education improves knowledge of anatomy and physiology, and physical exam skills 37, 38. The knowledge is retained 39. Additionally, it has been demonstrated that increased POCUS use leads to increased proficiency 40, 41, 42. This study shows that a good measure of competency can be achieved after a short course in POCUS. Extrapolating from the previous studies mentioned above 37, 38, 39, 40, 41, 42, it would be expected that the image acquisition ability and diagnostic accuracy demonstrated by the medical students on a wide range of POCUS scans in this study would only improve with continued education and practice, supporting the incorporation of formal POCUS education into medical school curricula. 

### Limitations

This study has several limitations. First, it was a small sample size of medical students who were recruited from an EM interest group who may have been more motivated to learn POCUS than the typical medical student.

Two bladder, 2 fracture, 15 cardiac and 18 IVC subjects were enrolled but unable to complete the study. Because the subjects were recruited during their stay in the pediatric emergency department with the goal of not interfering with patient care, it was assumed that the subjects could not complete the study due to ED care and not a medical student inability to obtain an image. We recognize, however, the possibility of a selection bias. Though unlikely, assuming all of the incomplete exams were due to an inability to obtain a proper image, the acceptable scan rate would decrease from 96.2% to 92.7% for bladder scans, 94.6% to 89.7% for the long bone scans, 96.7% to 85.9% for the cardiac scans, and from 84.6% to 73.3% for the IVC scans. 

The subjects undergoing cardiac and IVC scans were recruited from a group with low likelihood for pathology, with only 26/120 being at risk for volume depletion due to decreased oral intake, fever, vomiting, diarrhea, moderate anemia and orthostatic hypotension. This creates a potential interpretation bias that cardiac function would be expected to be normal and the IVC expected to not significantly collapse. 

Potential long bone scan subjects with fractures may have been less likely to consent for enrollment due to increased pain, and it is assumed, though not recorded by the medical student, that several subjects enrolled for long bone scans had radiographs completed and interpreted prior to enrollment in the POCUS study, thereby potentially affecting blinding of the medical student. 

## Conclusions

Medical students demonstrated proficiency in a range of POCUS scans on pediatric patients after a relatively short curriculum. This study provides more support for the feasibility of a successful short medical student POCUS curriculum, which should include pediatric patients. Future studies are needed to recommend a standardized medical student POCUS curriculum, as well as to evaluate for minimum numbers for competency and integration of medical student POCUS use in clinical practice.

## COI disclosure

None of the authors have any conflicts of interest to disclose.
